# Strong between‐site variation in New Caledonian crows’ use of hook‐tool‐making materials

**DOI:** 10.1111/bij.12757

**Published:** 2016-01-13

**Authors:** James J. H. St Clair, Barbara C. Klump, Jessica E. M. van der Wal, Shoko Sugasawa, Christian Rutz

**Affiliations:** ^1^ Centre for Biological DiversitySchool of BiologyUniversity of St AndrewsSt AndrewsKY16 9THUK

**Keywords:** construction behaviour, corvid, cumulative culture, extractive foraging, innovation, material culture, raw materials selectivity, tool manufacture, tool selectivity, tool use

## Abstract

Functional tool use requires the selection of appropriate raw materials. New Caledonian crows *Corvus moneduloides* are known for their extraordinary tool‐making behaviour, including the crafting of hooked stick tools from branched vegetation. We describe a surprisingly strong between‐site difference in the plant materials used by wild crows to manufacture these tools: crows at one study site use branches of the non‐native shrub *Desmanthus virgatus*, whereas only approximately 7 km away, birds apparently ignore this material in favour of the terminal twigs of an as‐yet‐unidentified tree species. Although it is likely that differences in local plant communities drive this striking pattern, it remains to be determined how and why crows develop such strong site‐specific preferences for certain raw materials.

## Introduction

Differences in behaviour between interconnected animal populations are of considerable interest to behavioural and evolutionary ecologists because they can indicate the action of powerful selective forces or cultural biases (Kawecki & Ebert, 2004; Laland & Janik, 2006; Lycett, Collard & McGrew, 2010). Close inspection often reveals that a combination of ecological variation and behavioural plasticity is sufficient to create striking landscape‐level patterning in phenotypic expression (Laland & Janik, 2006). In the present study, we report evidence of marked between‐site variation in the materials used by New Caledonian (NC) crows, *Corvus moneduloides*, to make one of their most complex foraging tools: hooked stick tools.

NC crows are known to manufacture tools from a wide range of plant materials, including leaves, grass stems, fern stolons, leaf petioles, and stem sections of various tree and vine species (Hunt, 1996, 2008; Troscianko, Bluff & Rutz, 2008). Their tools can be classified into those excised from the margins of screw‐pine leaves (pandanus tools) and those made from parts of other plants (stick‐type tools), with the latter subclassified into hooked and nonhooked stick tools (Rutz & St Clair, 2012). Growing evidence suggests that NC crow populations use different plant materials to make tools of a given type. For example, birds at some sites predominantly use the leaf petioles of candlenut trees *Aleurites moluccana* as nonhooked tools with which to ‘fish’ for woodboring cerambycid beetle larvae (Hunt, 2000), whereas, at others, a more diverse range of materials is used for the same function (Bluff *et al*., 2010b). Moreover, raw materials usage can change over time, at least at the population level: the anthropogenically‐introduced vine species *Lantana camara* has become the favoured raw material for tool manufacture at several sites (Hunt, 2008).

**Figure 1:**
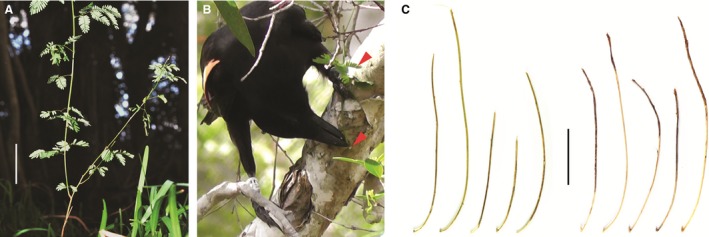
A, *Desmanthus virgatus* growing in the wild (scale bar = 5 cm). B, still image (from video) of a colour‐marked wild crow (ID ring code ‘ER4’) engaged in tool manufacture from *D. virgatus*. The crow is grasping the hooked end of a twig (lower arrow) prior to ‘crafting’ it further. The distinctive pinnate leaves of *D. virgatus*, which the crow has not yet removed, are evident just above its left foot (upper arrow). C, sample of representative tools recovered from wild crows at two sites approximately 7 km apart. Left: five tools made from *D. virgatus*, from the farmland site. Right: five tools made from the terminal twigs of an unknown tree species, from the beach site (scale bar = 5 cm).

We discovered that crows at one of our study sites almost exclusively use stems of a non‐native but widespread shrub species, *Desmanthus virgatus* (Fig. 1A), as the raw material for hooked stick tool manufacture. Unexpectedly, crows living only approximately 7 km away, and which have access to the same plant material, appear to largely or wholly shun it in favour of other plant species, providing a striking example of regional variation in tool‐making preferences. The local availability of raw materials is likely to contribute to this pattern, although the role of individual and social learning in the development of crows’ materials usage, and in particular the possibility that their preferences are culturally influenced, merits further investigation.

## Material and methods

### Study sites

Research was conducted from 2011 to 2014 at two study sites on the central west coast of Grande Terre, New Caledonia (for a map and further details, see Rutz, Ryder & Fleischer, 2012): a farmland area in the Gouaro‐Déva reserve, and a nearby residential beachside area. As detailed below, we used a suite of field methods to document crows’ usage of raw materials for making hooked stick tools (for a similar approach with chimpanzees, see Sanz, Morgan & Gulick, 2004; Sanz & Morgan, 2007).

### Field observations

#### Farmland site

Following an initial opportunistic observation of hooked stick tool manufacture at this site (2011), a combination of opportunistic and systematic observations were made in 2013–2014. Systematic observations took the form of ‘focal follows’, where crows were located by fieldworkers walking set transect routes, and followed at a distance of approximately 5–30 m. Tool manufactures and other foraging‐related behaviours were filmed using hand‐held video cameras (Sanyo Xacti VPC‐CA9; Panasonic V700; Panasonic SD900) and, where possible, tool materials were identified by visual inspection of discarded trimmed leaves and stem sections. On‐going analysis suggests that tool‐derived prey at the farmland site covers a wide range of sizes and taxonomic groups.

#### Beach site

Because crows at this site range across multiple private properties, the following of focal subjects was impractical and, instead, birds were observed in a single garden by two means: opportunistically during regular site visits; and with motion‐triggered video cameras (Bushnell Trophy Cam HD; Bluff *et al*., 2010b) installed near a water bath where local birds occasionally arrived with their foraging tools, to drink or bathe (see below).

### Tool collection

#### Both sites

Tools were collected opportunistically in 2012–2014. Most were recovered by the ‘distraction’ method, where an attractive nontool‐acquired resource (meat scraps or a water bath) was provided to crows, and the surrounding area periodically checked for any tools that had been cached or abandoned by visiting birds. Others were recovered from crows using the ‘startle’ method (Hunt, 1996), in which the researcher tries to make the crow drop its tool by making a sudden movement or loud noise.

### Behavioural experiments

#### Farmland site

Crows were captured with meat‐baited whoosh‐net traps (in which a net is pulled over ground‐feeding birds using pre‐tensioned elastic cords; Gosler, 2004) in 2012 and 2013. Of 41 trapped crows, one escaped from the aviary and eight were released either immediately or within 2 days (as a result of breeding status or health issues); two birds released upon capture in 2012 were re‐captured and used in experiments in 2013. The 34 resulting subjects were housed individually, apart from adults trapped with dependent young. Water was available at all times, and food was only removed for behavioural trials (for husbandry details, see St Clair & Rutz, 2013). Trials took place in a separate experimental aviary and were observed from an adjoining blind. To encourage tool‐manufacture behaviour, we presented a ‘food log’ containing drilled holes baited with meat, and candidate tool materials wedged into holes of a ‘materials log’ (Klump *et al*., 2015). Two of the first‐caught birds were presented with locally‐sourced stems from a range of plant species (including *D. virgatus*) that appeared to be suitable candidates for hooked stick tool making (apart from paperbark twigs *Melaleuca* sp., we were unable to identify these plants). Given that only *D. virgatus* was chosen by subjects in these early trials, and that observation of wild birds showed that this species was overwhelmingly selected, we subsequently provided only *D. virgatus* to crows both in their housing aviaries (34 crows) and during behavioural trials (29 crows).

#### Beach site

We trapped nine crows in this area, also with whoosh‐nets, in 2013 (between‐site differences in sample size were largely a result of differences in trapping effort). Four birds were released before taking part in any trials because of their breeding or health status. The remaining five birds were housed as described above, and included a bonded pair that was both housed and tested together. Subjects were tested two to 11 times, as described above, with various materials presented on the ‘materials log’. *Desmanthus virgatus* was presented to all crows at the beach site during trials and in their housing aviaries, whereas other candidate materials were tested as and when we found them, leading to birds experiencing varying choices and numbers of trials (only *D. virgatus*: two birds, one trial each; *D. virgatus* and other local plant stems: four birds, two to three trials each; *D. virgatus* and assorted twigs and leaf petioles: four birds, two to four trials each; *D. virgatus*, other local plant stems and assorted twigs and leaf petioles: four birds, one trial each). Trials were recorded either by an observer with a camcorder from an adjoining blind, or with an (unattended) motion‐triggered video camera (Bushnell Trophy Cam HD).

## Results

### Field observations

On 6 November 2011 in the farmland site, an adult male crow (ID ring code ‘EK1’) was observed foraging on the ground in short pasture, at a distance of approximately 40 m. The crow probed briefly with a tool of unknown type, possibly a grass stem. It then flew 20–30 m into a shrubby area, harvested a branched stem from a plant close to the ground, and flew with this stem up to a low perch where it proceeded to make a tool by removing leaves and stem sections. It then appeared to manipulate the functional end of the tool; the entire process took 1–2 min. Although the shape of the tool was not clearly visible, the actions resembled the latter stages of hooked stick tool manufacture (removal of leaves and side branches, and crafting of the hook; Hunt & Gray, 2004; Klump *et al*., 2015). Once manufacture was completed, the crow flew back to the original foraging location and, after a few seconds of probing in the ground with its new tool, withdrew it with a dark‐coloured prey item, approximately 2 cm long, attached to the end. The crow ate the prey, and flew away with the tool when approached. The soil in the probe site contained a large, vertical silk‐lined burrow, presumably that of a trap‐door spider, with no apparent occupant. Inspection of the area where the crow had previously perched yielded fresh trimmings of *D. virgatus*.

During subsequent fieldwork (2013–2014) at the same study site, 39 further tool manufactures of hooked stick tools were observed, of which 37 were by five different wing‐tagged crows (Fig. 1B) and two were by unmarked birds. Among these manufactures, there were 27 that were completed and in which we could see the material sufficiently well to identify or rule out certain plant species. Twenty‐three (85%) of these manufactures used *D. virgatus* (18 confirmed; five probable), one (4%) used an unknown material that was clearly not *D. virgatus*, and three (11%) used paperbark (*Melaleuca* sp.) twigs. Six tool manufactures were not completed (i.e. the crow initiated hooked stick tool manufacture but abandoned the tool before reaching the hook‐crafting stage): two of these used an unknown material that was clearly not *D. virgatus*, a further two used paperbark twigs, and two used *D.  virgatus*, suggesting that manufactures from non‐*Desmanthus* materials were less likely to be completed (χ^2^_ _= 4.64, d.f. = 1, *P *=* *0.031).

At the beachside area, opportunistic observations and footage from motion‐triggered video cameras indicated that at least seven different crows (identifiable either from colour‐rings or distinctive characteristics) regularly visited the garden where two water baths had been placed for tool collection. Several birds were seen carrying tools, including hooked stick tools. A single case of tool manufacture from live plant material was observed on 22 September 2012, although this involved the production of a nonhooked stick tool.

### Recovered tools

Over the period 2012–2014, 16 hooked stick tools were opportunistically collected from wild NC crows at the farmland site; in nine cases, the tool's user was not seen, whereas at least four different crows accounted for the remaining seven tools (six were recovered from three wing‐tagged individuals, and one from an unmarked bird). The raw material was identifiable as *D. virgatus* in 11 of the 16 tools (69%) (Fig. 1C), whereas the raw material of the remainder was consistent with *D. virgatus* but could not be confidently identified.

At the beach site over the same period, 53 abandoned hooked stick tools were recovered at the water baths (some nonhooked tools were also retrieved but are not discussed here). For at least 51 of the hooked stick tools (96%), we could rule out *D. virgatus* and, in many cases, it was clear that tools were made from terminal branches of the same tree species (Fig. 1C); despite wide‐ranging searches of the study area, and despite consulting several local botanists and other experts, we have not yet been able to identify the species.

### Behavioural experiments

Of 34 farmland crows presented with *D. virgatus*, either in their housing aviary or in behavioural trials, 27 produced at least one hooked stick tool from this material. Of the two farmland crows presented with a choice of raw materials, both manufactured tools exclusively from *D. virgatus* (a preference that has subsequently been confirmed with additional subjects in separate experiments). Whether provided only with *D. virgatus* or given a choice between *D. virgatus* and alternative locally‐sourced materials, none of the birds at the beach site manufactured hooked stick tools from provided materials, although two made nonhooked stick tools (from *D. virgatus* and other unidentified material) and several used supplied (nonhooked) sticks that did not require processing.

## Discussion

We found that NC crows at our farmland study site have a strong tendency to use a single introduced shrub species as the raw material for hooked stick tool manufacture, whereas crows at a beachside area only approximately 7 km away largely or entirely ignore this plant in favour of alternative materials. Given that *D. virgatus*, which is native to the neotropics (Luckow, 1993), was probably introduced to New Caledonia subsequent to European colonization in the mid‐19th Century, it follows that the population at the farmland site has either switched from other materials, or even started making hooked stick tools in the relatively recent past. Indeed, during fieldwork at this site in 2005–2008, only the use of nonhooked stick tools had been documented (twigs and grass stems: Rutz *et al*., 2007; Troscianko *et al*., 2008), raising the intriguing possibility that hook making may have been rare or absent from this population until a few years ago. Such a sudden change in local tool‐making habits could have resulted from the immigration of crows from hook‐making stock; for example, from a dry‐forest area approximately 10 km away (first hooked stick tool observations in 2007, L. Bluff and C. Rutz, unpubl. data; video evidence from miniature crow‐mounted cameras in 2010, Troscianko & Rutz, 2015).

Together with earlier work, our findings show that: there is marked between‐site variation in the plant species used by NC crows for hooked stick tool manufacture (Hunt, 1996; present study); such differences in materials usage can occur over surprisingly short distances of just a few kilometres (present study); within‐site choice of plant species for tool manufacture may range from variable (Hunt, 1996) to relatively invariant (Hunt & Gray, 2004; present study); population‐level change can occur over a relatively short timescale of less than approximately 150 years (Hunt, 2008; present study); and at least some individuals exhibit flexibility in material choice (crow ‘ER4’ was observed making tools in the wild from both *D. virgatus* and *Melaleuca* sp.; present study). These patterns, as well as the fact that some long‐term captive NC crows will readily make tools from novel materials (Weir & Kacelnik, 2006), strongly suggest that learning plays a role in materials selectivity by wild NC crows. Even so, an element of local adaptation cannot be entirely ruled out; a recent analysis showed that crow populations at the two sites investigated in the present study, which make use of different materials, are also significantly genetically differentiated despite their close proximity (Rutz *et al*., 2012).

Between‐site variation in an important component of NC crows’ tool‐use behaviour (i.e. the selection of raw materials) is of considerable interest given the proposed existence of cumulative technological culture in this species (Hunt & Gray, 2003). Cultural inheritance requires that individuals learn socially (from each other) rather than purely individually (through processes including trial‐and‐error). Despite considerable speculation, the relative importance of individual and social learning for any aspect of NC crows’ tool‐related behaviour remains unknown (Bluff, Kacelnik & Rutz, 2010a; Logan *et al*., 2015). If, however, crows socially learn which materials to use in tool manufacture, the differences in materials usage described in the present study may reflect cultural variation over a remarkably small spatial scale; our two study sites are only a few thousand metres apart ‘as the crow flies’ (such local‐scale variation in tool‐related behaviour is infrequently described; for another recent example, see Koops *et al*., 2015). The persistence of local traditions over such short distances would implicate either restrictions in the diffusion of information (such as a highly modular social structure; St Clair *et al*., 2015), powerful cultural mechanisms promoting conformity within sites (Aplin *et al*., 2014) or ecological differences between sites that favour corresponding (socially or individually learned) differences (see below).

The fact that birds within a given site tend to converge on the same material would imply that their learning processes are very consistent, or at least lead to consistent outcomes. If individual learning is important, the pattern would suggest that, during the process of developing materials preferences, individuals are guided by pre‐existing biases that cause them to discover and adopt, out of the dozens or hundreds of options available at a given site, materials with highly specific properties. Informal examination of hooked stick tools manufactured at both of our sites suggests that materials indeed comprise a nonrandom sample of the available options with respect to a number of properties. Apparent commonalities include a small absolute stem diameter (Fig. 1C), relatively high ratios of both strength and rigidity to stem diameter, the presence of relatively acutely‐angled forks, noticeable stem curvature, lack of a weak ‘fracture point’ in the fork itself, and the absence of highly poisonous or sticky sap. Materials selectivity is not limited to the choice of plant species. Recent experimental work has shown that the farmland crows choose among *D. virgatus* stems according to their properties, preferring stems of intermediate robustness, which are neither too difficult to detach from the plant, nor too flimsy to function effectively (Klump *et al*., 2015). In other nonhuman tool users, material preferences have so far been linked to a maximum of three unidimensional properties (weight, volume, and friability of rocks used by capuchin monkeys as hammers; Schrauf, Huber & Visalberghi, 2008; Visalberghi *et al*., 2009). The ability of NC crow individuals (or groups) to identify materials with highly specific mechanical properties may be a potentially vital component of their remarkable tool‐related skills, and could help to explain why this species, apparently alone with humans, expresses hooked tool manufacture in the wild.

Turning our attention to ultimate explanations for the observed pattern, there are a number of reasons that different materials might be used in different locations. One is that the variation could result from (genetic or cultural) drift processes, and thus be simply random. Another is that differences might reflect adaptation to local foraging challenges, with different materials used to make tools that are suited to different tasks; for example, for targeting different prey types and/or extraction contexts. This hypothesis is not well supported by current evidence: hooked stick tools made at the two study sites are generally of similar size and shape (Fig. 1C) and crows at the farmland site acquire a relatively wide range of prey with their hooked stick tools. A more compelling explanation for the observed pattern is that crows’ choices are simply constrained by the availability of raw materials (for a classic investigation of habitat effects on chimpanzee tool use, see McBeath & McGrew, 1982); in other words, if only a small fraction of the locally distinct community of plants is suited to the manufacture of a particular tool type, their discovery, selection, and exclusive use by resident crows can result in highly site‐specific patterns of materials usage. Consistent with this latter interpretation, there are notable differences in floristic composition and diversity between the two sites in the present study, with relatively species‐poor degraded pasture and open woodland in the farmland site, and a relatively species‐rich mixture of native dry forest, mangrove swamps, and ornamental gardens at the beach area. Formal comparison of the abundance and properties of candidate hook‐making material remains a challenge for the future, although our preliminary surveys confirm that *D. virgatus* is present and accessible at both sites (but may be more abundant at the farmland site). The reluctance of the beach crows to use *D. virgatus* raises the possibility that the material is in some way inferior to the locally‐preferred one, and that the exclusive use of *D. virgatus* at the farmland site may be linked to the absence of functionally equivalent or superior alternatives. In crows (as in other species such as chimpanzees: Lycett *et al*., 2010; Koops *et al*., 2015), further work is required to clarify both the ultimate drivers and proximate mechanisms that produce regional differences in tool‐making habits.
